# Recruitment of Cbl-b to B Cell Antigen Receptor Couples Antigen Recognition to Toll-Like Receptor 9 Activation in Late Endosomes

**DOI:** 10.1371/journal.pone.0089792

**Published:** 2014-03-20

**Authors:** Margaret Veselits, Azusa Tanaka, Stanley Lipkowitz, Shannon O'Neill, Roger Sciammas, Alison Finnegan, Jian Zhang, Marcus R. Clark

**Affiliations:** 1 Section of Rheumatology, Department of Medicine and Knapp Center for Lupus and Immunological Research, University of Chicago, Chicago, Illinois, United States of America; 2 Laboratory of Cellular and Molecular Biology, Center for Cancer Research, National Cancer Institute, National Institutes of Health, Bethesda, Maryland, United States of America; 3 Integrated Department of Immunology, National Jewish Medical and Research Center and University of Colorado and Health Sciences Center, Denver, Colorado, United States of America; 4 Department of Immunology and Microbiology, and Department of Internal Medicine, Section of Rheumatology, Rush University Medical Center, Chicago, Illinois, United States of America; 5 Section of Nephrology, Department of Medicine, University of Chicago, Chicago, Illinois, United States of America; University of Miami, United States of America

## Abstract

Casitas B-lineage lymphoma-b (Cbl-b) is a ubiquitin ligase (E3) that modulates signaling by tagging molecules for degradation. It is a complex protein with multiple domains and binding partners that are not involved in ubiquitinating substrates. Herein, we demonstrate that Cbl-b, but not c-Cbl, is recruited to the clustered B cell antigen receptor (BCR) and that Cbl-b is required for entry of endocytosed BCRs into late endosomes. The E3 activity of Cbl-b is not necessary for BCR endocytic trafficking. Rather, the ubiquitin associated (UBA) domain is required. Furthermore, the Cbl-b UBA domain is sufficient to confer the receptor trafficking functions of Cbl-b on c-Cbl. Cbl-b is also required for entry of the Toll-like receptor 9 (TLR9) into late endosomes and for the *in vitro* activation of TLR9 by BCR-captured ligands. These data indicate that Cbl-b acts as a scaffolding molecule to coordinate the delivery of the BCR and TLR9 into subcellular compartments required for productively delivering BCR-captured ligands to TLR9.

## Introduction

Antigen presentation by B lymphocytes is required to mount high affinity humoral immune responses, for coordinating antigen specific cytotoxicity, and for propagating some T cell responses [1]. B lymphocytes differ from other antigen presenting cells in several fundamental ways. The most important difference is that B cells are clonotypic, and they usually only efficiently capture and process antigens recognized by the B cell antigen receptor (BCR) [2]. The primacy of the BCR as the portal for entry of antigen ensures coordination of B and T cell responses.

In B cells, most antigens are processed in specialized MHC class II containing late endosomes (MIIC) [3] which are Lamp-1^+^, acidic and contain cathepsins, thiol reductases, and other molecules required for efficient antigen processing [4]. MIIC vesicles consist of a limiting membrane studded with Lamp-1 and a lumen containing multivesicular bodies [5]. These intraluminal vesicles are derived from BCR-laden transport vesicles that have gained access to the MIIC compartment [6].

BCR trafficking to late endosomes is also required for coupling antigen recognition to the activation of the toll-like receptors (TLRs) 7 and 9 [7], [8]. This is because these receptors only productively bind ligands in late endosomes. The mechanisms underlying this requirement have been best defined for TLR9. In resting B cells, TLR9 resides outside the MIIC. Upon BCR ligation, TLR9 rapidly transits into the MIIC [9], [10] where the receptor can bind DNA containing complexes captured by the endocytosed BCR [11]–[13]. Analysis of BCR and TLR9 endocytic trafficking in anergic B cells, in which the trafficking of both receptors is aberrant, indicates that entry of the BCR and TLR9 into late endosomes is coordinated and that both receptors enter on common transport vesicles [10]. Presumably, this facilitates the transfer of BCR captured ligands to the TLRs.

Work from several laboratories has provided a general model for how endocytosed receptor complexes are sorted through early endosomes and delivered into late endosomal multivesicular bodies [14]. Central to this model is the monoubiquitination of receptors and the recognition of these ubiquitins by a protein complex containing Hrs, Eps15 and STAM (the endosomal complex required for transport, ESCRT-0). ESCRT-0 engaged receptors are retained within the endosomal pathway while unbound receptors recycle to the cell surface. Successive recruitment of the multimeric complexes ESCRT-I, ESCRT-II, and ESCRT-III target receptors to late endosomes. These receptors are then sorted into intraluminal multivesicular bodies where they are degraded. While the ESCRT complexes constitute the core machinery for the delivery of receptors to late endosomes, several other molecular complexes are involved in facilitating and regulating ESCRT-mediated endocytic transit [15].

Previously, we have demonstrated that the BCR subunit Igβ is ubiquitinated and that this is required for sorting to late endosomes [16]. Normal receptor ubiquitination required Itch, a member of the Nedd4 family of E3s. This is in apparent contrast to the T cell receptor (TCR) [17], and other receptors [15], where recruitment of the Casitas B-lineage Lymphoma (Cbl) E3s to the tyrosine phosphorylated receptor induce ubiquitination. We now report that Cbl-b is also required for BCR endocytic trafficking, and that it contributes to receptor ubiquitination following receptor stimulation. However, Cbl-b ligase activity is dispensible for BCR endocytic trafficking. Rather, Cbl-b provides a necessary scaffolding function that is dependent upon the carboxyterminal tail. Surprisingly, transit of TLR9 into late endosomes was also dependent upon Cbl-b. These and other findings demonstrate a unique, unexpected, and functionally important role for Cbl-b in directing the delivery of both the BCR and TLR9 to late endosomes.

## Materials and Methods

### Mice

Wild-type, *Cblb^−/−^* (Balb/c), and *Cblb^C373A^* (C57BL/6J) [18] mice were housed in the animal facilities of the University of Chicago. Mice were used at 8 to 12 weeks of age, and experiments were in accordance with the guidelines of the Institutional Animal Care and Use Committee of the University of Chicago (Protocol Number: 71577, approval date 4/6/13). All animal studies were carried out in strict accordance with the recommendations in the Guide for the Care and Use of Laboratory Animals of the National Institutes of Health. All mice were sacrificed by CO_2_ inhalation followed by cervical dislocation.

### Cells and culture conditions

Splenic B cells were isolated by negative selection using biotinylated anti-CD11b (M1/70), anti-CD11c (HL3), anti-NK1.1 (PK136), anti-Ter-119, anti-CD3ε1452C11, anti-CD4 (RM4-5), anti-CD8α (53-6.7), anti-Ly-6G, and Ly-6C (RB6-8C5, all BD Bioscience) followed by streptavidin magnetic beads (MACS; Miltenyi Biotec) [16]. Cells were cultured for 20 hours in complete DMEM supplemented with 10% FCS, 50 ng/ml rIL-4 (R & D Systems), and 7.5 µg/ml anti-CD-40 (BD Biosciences).

### Receptor internalization and ubiquitination assays

BCR internalization assays on wild-type and *Cblb^−/−^* splenic B cells were performed as previously described [19]. Receptor ubiquitination assays were performed as described [16].

### Retroviral gene transduction

The cDNA encoding human Cbl-b and truncation mutants as well as human c-Cbl [20] were subcloned into the plasmid MIGR1. Calcium Phosphate transfection of PlatE cells with MIGR1 was carried out as previously described [21], [22]. Aliquots of cell lysates were immunoblotted to confirm the size of the expressed proteins. Cbl-b (H-454 and H-121) and c-Cbl (C-15) antibodies were from Santa Cruz Biotechnology. Wt and *Cblb^−/−^* B cells were transduced by resuspending in viral supernatant with 8 mg/ml polybrene in the presence of rIL-4 (50 ng/ml) and anti-CD40 (7.5 µg/ml) and centrifuging at 1000× g for 1.5 hr at room temperature. The cells were then washed and placed into fresh culture medium and allowed to grow for 48 hrs.

### Confocal microscopy

Confocal Microscopy was performed as previously described [10]. Images were collected by using a Leica TCS SP2 AOBS confocal microscope. Antibodies used for visualization were Lamp-1 (1D4B) (BD Biosciences), TLR9 (26C593.2) (Imgenex), Cathepsin L (CPLH-3G10) and Cbl-b (H-121 and H-454) (Santa Cruz Biotechnology). All secondary antibodies were Alexa Fluor conjugated IgG (H & L) from Invitrogen. To quantitate the extent of colocalization between 2, 3 or 4 fluorescent markers in individual cells, we used the JACoP plug-in of ImageJ. The specific algorithm used was based on the Mander's coefficient with a threshold of 40 [23]. Cells that had a Mander's coefficient of 0.25 or higher were scored as positive for co-localization. For each experiment, at least 30 randomly selected cells were scored.

### T-bet assay and quantitative real-time PCR

Biotinylated ODN 1826 and biotinylated control ODN were from Invivogen. Purified splenic B cells from WT and *Cblb^−/−^* mice were incubated with 10 µg/ml of streptavidin-conjugated F(ab)_2_ Ig (H & L) followed by either the biotinylated ODN 1826 or control ODN (Invivogen). Cells were then incubated for 6 hrs at 37°C. RNA was isolated and quantitative PCR performed as previously described [10].

### Western blotting and immunoprecipitations

Splenic B cells were purified by negative selection as described above. Cells were stimulated with 20 µg/ml F(ab)_2_ goat anti–mouse Ig (H+L) (Jackson ImmunoResearch Laboratories) at 37°C for indicated times. Cell aliquots were lysed in 1% NP40 buffer (150 mM NaCl, 10 mM Tris HCl pH 7.7, 5 mM EDTA, 0.4 mM sodium orthovanadate, and 10 mM sodium pyrophosphate) containing mini EDTA-free protease inhibitor cocktail tablets (Roche), phenylmethylsulfonyl fluoride (PMSF)(Sigma-Aldrich), 20 mM N-Ethylmaleimide (NEM) (Sigma-Aldrich), 10 mM 1,10-phenatholine monohydrate (OPD) (Sigma-Aldrich), and 50 µM PR-619 (LifeSensors). Lysates were clarified by centrifugation at 4°C. Lysates were precleared with protein A–Sepharose (Pierce), incubated with primary antibodies specific for Igβ (Hm79b, BD Biosciences) and captured with protein G–Sepharose (Pierce). Lysates or immunoprecipitates were resolved on a 4–15% Mini-Protean TGX gel (Bio-Rad) and transferred onto Immun-Blot PVDF membrane (Bio-Rad). Membranes were probed with antibodies specific for ubiquitin (P4D1, Santa Cruz), Igβ [24] or Cbl-b.

### Statistical Analyses

Statistical comparisons were made using the Student's t-test.

## Results

### Cbl-b is required for BCR endocytic trafficking to late endosomes

**Figure 1:**
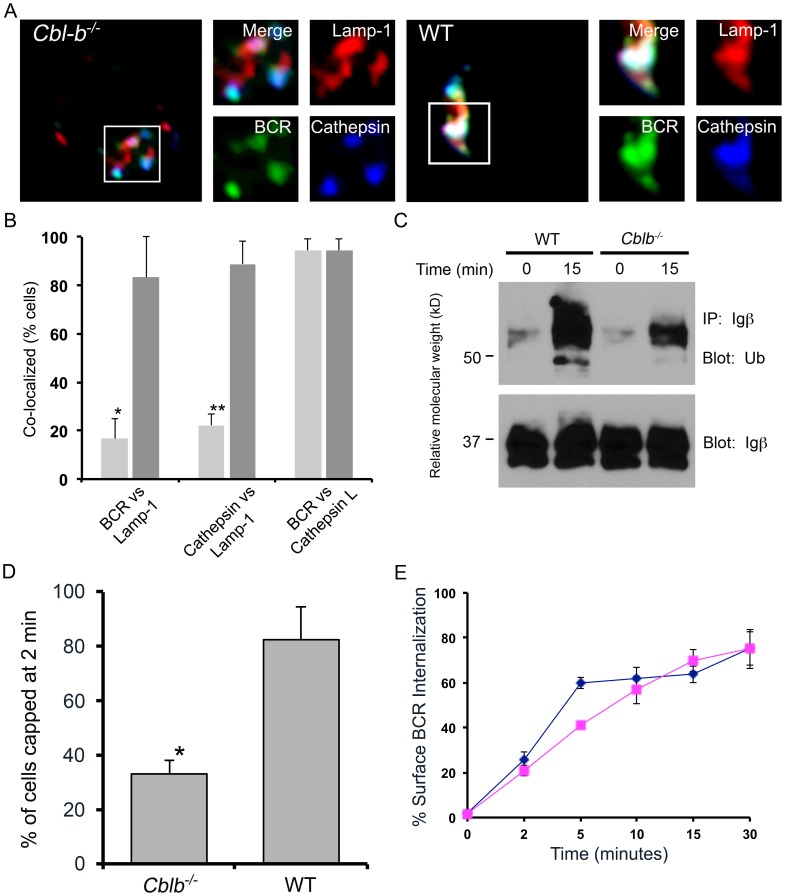
Cbl-b is required for normal trafficking to late endosomes. (A–B) Trafficking to Lamp-1^+^ and Cathepsin L^+^ endocytic compartments by the ligated BCR in WT and *Cblb^−/−^* splenic B cells. (A) Representative confocal images of BCR stimulated cells (30 min) and (B) quantitative analysis of three independent experiments (30 cells/exp) (*p = 0.0003 and **p = 0.0004). Average Mander's coefficient of BCR vs Lamp-1 for WT cells was 0.556±0.12 vs 0.146±0.07 for *Cblb^−/−^* cells (p<0.0001). Average Mander's coefficient of Cathepsin-L vs Lamp-1 for WT cells was 0.415±0.05 vs 0.179±0.03 for *Cbl-b^−/−^* cells (p<0.002). (C) Ubiquitination of Igβ in *Cblb^−/−^* and WT splenocytes. Unstimulated or BCR stimulated splenocytes were lysed, immunoprecipitated with anti-Igβ antibodies, and then sequentially immunoblotted with anti-Ub (upper panel) or anti-Igβ (lower panel) antibodies. Results are representative of three experiments. (D) Cells were stimulated as in “A” for two minutes, fixed, and visualized by confocal microscopy. Shown are the percentages of each cell population that formed a receptor cap on the cell surface containing more than 50% of visualized BCRs (n = 3 experiments, p = 0.005). (E) Internalization of BCR in *Cblb^−/−^* (blue, diamonds) and WT (purple, squares) splenocytes in response to anti-IgM F(ab)_2_ antibodies. Results are representative of three experiments.

To explore if Cbl-b played a role in BCR endocytic trafficking, splenic B lymphocytes [10] from wild type (WT) or *Cblb^−/−^* Balb/c mice were isolated and then stimulated *in vitro* through the BCR with FITC-conjugated anti-Ig F(ab)_2_ antibodies for 30 minutes at 37°C. Aliquots were then fixed, stained with anti-Lamp-1 antibodies (Alexa Fluor 647, Invitrogen), and visualized by confocal microscopy [10]. Representative images are provided in **Figure 1A**, and a quantitative analysis of the fraction of cells demonstrating significant overlap (>25%) between the BCR and Lamp-1 is provided in **Figure 1B** [10]. In WT cells, the BCR rapidly targeted Lamp-1^+^ late endosomes with almost all cells demonstrating strong co-localization between the BCR and Lamp-1. In contrast, in *Cblb^−/−^* cells there was little co-localization between the BCR and Lamp-1. Rather, the endocytosed BCR was in close proximity with Lamp-1^+^ late endosomes. Similar results were obtained if cells were stimulated for 60 minutes (data not shown).

We hypothesized that in the absence of Cbl-b, endocytosed BCRs might target terminal lysosomes which in B lymphocytes, do not contain Lamp-1 [25]. As demonstrated in **Figures 1A and 1B**, the Lamp-1^−^ subcellular compartment targeted by the BCR in *Cblb^−/−^* splenocytes contained cathespin L. Interestingly, Cathepsin L was much more apparent in the Lamp-1^+^ compartments of WT than *Cblb^−/−^* splenocytes. This latter observation suggests that Cbl-b lies in the signaling pathway necessary for BCR-mediated MIIC maturation [10], [26], [27].

To determine if Cbl-b contributed to BCR endocytic trafficking by ubiquitinylating Igβ, splenic B lymphocytes from WT or *Cblb^−/−^* mice were stimulated with anti-Ig F(ab)_2_ antibodies for 15 minutes at 37°C. Aliquots were then lysed in a buffer containing 1% NP40, and lysates were precipitated with anti-Igβantibodies. Precipitations were resolved by SDS-PAGE, transferred to PVDF membranes, and probed with anti-ubiquitin (Ub) antibodies. As can be seen in **Figure 1C**, there was approximately a three-fold decrease in the amount of ubiquitinated Igβ immunoprecipitated from *Cblb^−/−^* B cells following BCR stimulation. These results indicate that Cbl-b, along with the E3 ligase Itch [16], contributes to inductive Igβ ubiquitination.

Recently, in chicken B cell lines, Cbl has been demonstrated to be required for ligand-induced BCR clustering on the cell surface [28]. Therefore, cells were stimulated as above for two minutes and then visualized by confocal microscopy. As demonstrated in **Figure 1D**, while more than 80% of WT B cells formed a cap following BCR stimulation, only approximately 35% of *Cbl-b^−/−^* B cells formed caps (p = 0.005). These data indicate that Cbl-b is required for BCR clustering in primary murine splenocytes.

Despite the difference in capping, subsequent internalization of the ligated BCR on both WT and *Cblb^−/−^* splenic B cells were similar (**Figure 1E**). Furthermore, transit of the ligated BCR to EEA1^+^ early endosomes was similar in WT and *Cblb^−/−^* splenic B cells (**Figure S1**) [10]. However, *Cblb^−/−^* BCRs left this compartment much more rapidly that WT BCRs. These data indicate that Cbl-b is specifically required for two discrete processes, BCR clustering and trafficking between early and late endosomes.

### Rapid recruitment of Cbl-b following BCR stimulation

**Figure 2:**
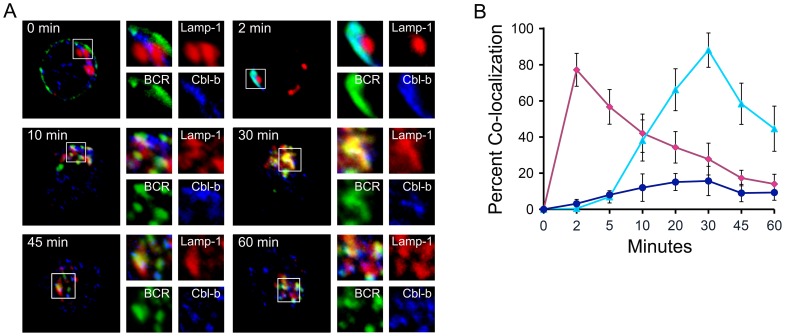
Cbl-b is recruited to the BCR and co-traffics to late endosomes. WT splenic B cells were stimulated through the BCR for the indicated times. (A) Representative confocal images demonstrating relative locations of BCR (green), Lamp-1 (red), and Cbl-b (blue) over time. (B) Quantitative analysis (n = 3, 30 cells/exp for each condition and time point) of co-localization over time of BCR and Cbl-b (diamond, red), BCR and Lamp-1 (triangle, light blue), and Cbl-b and Lamp-1 (circle, dark blue).

To examine if Cbl-b directly participated in BCR endocytic trafficking, we first determined if Cbl-b was recruited to the BCR following receptor engagement. Splenic WT Balb/c B lymphocytes were stimulated as above with Texas Red-conjugated anti-Ig F(ab)_2_ antibodies for various times up to 60 minutes. Cells were then fixed, stained with anti-Lamp-1 (Alexa Fluor 647) and anti-Cbl-b antibodies (Alexa Fluor 488, Invitrogen), and visualized by confocal microscopy. Representative images are provided in **Figure 2A** with the corresponding quantitations from three independent experiments provided in **Figure 2B**. As can be seen, Cbl-b was rapidly and almost completely recruited to the BCR within two minutes of ligation. The BCR and Cbl-b remained co-localized as the receptor was internalized and targeted to late endosomes. However, the association between the BCR and Cbl-b diminished as the BCR started to enter Lamp-1^+^ late endosomes. These data indicate that Cbl-b is rapidly and robustly recruited to the BCR and then traffics with the receptor through the endocytic pathway. However, the majority of Cbl-b does not enter Lamp-1^+^ late endosomes with the BCR.

### The Cbl-b UBA domain, but not the RING finger domain, are required for BCR endocytic trafficking

**Figure 3:**
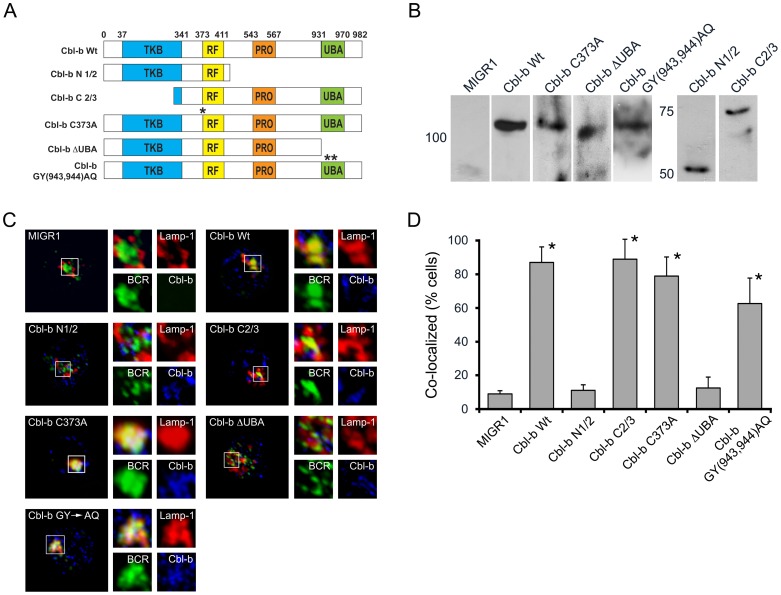
BCR endocytic trafficking is dependent on the UBA domain of Cbl-b and independent of its ligase activity. (A) Schematic representation of different Cbl-b mutants that were used to reconstitute Cblb*^−/−^* splenocytes (TKB, tyrosine kinase binding domain; RF, ring finger domain; PRO, proline rich regions; UBA, ubiquitin binding domain). Numbers above schematic refer to amino acid positions. (B) Immunoblots of packaging cell lysates with anti-Cbl-b antibodies demonstrating the relative molecular weights of the indicated expressed Cbl-b mutants. (C) Representative confocal images demonstrating the ability of each indicated Cbl-b mutant to reconstitute BCR endocytic trafficking in *Cblb^−/−^* splenocytes. For these experiments, cells were stained with anti-BCR (green), anti-Lamp-1 (red), and anti-Cbl-b antibodies (blue). (D) Quantitative analysis (n = 3, 30 cells/exp for each condition) for co-localization of BCR with Lamp-1 (* statistically similar, p<0.001 for Cbl-b WT vs. N1/2, ΔUBA).

We next examined which functional domains of Cbl-b were required for BCR endocytic trafficking. For these experiments, cDNAs encoding WT Cbl-b and a series of Cbl-b mutants (**Figure 3A**) [20], [29] were cloned into the retroviral vector MIGR1. These vectors were then packaged in PlatE cells [21]. Aliquots of cell lysates from packaging cells were immunoblotted to confirm expression of Cbl-b proteins of the expected relative molecular weights (**Figure 3B**). Splenic B cells were harvested from *Cblb^−/−^* mice and cultured for 24 hours in IL-4 (50 ng/ml) and anti-CD40 antibodies (7.5 µg/ml). Aliquots of proliferating *Cblb^−/−^* splenic B cells were then infected with viruses encoding the indicated Cbl-b molecules. After an additional 48 hours, cells were stimulated with Texas Red conjugated anti-BCR F(ab_2_) antibody fragments for 30 minutes, then fixed and stained with anti-Lamp-1 and anti-Cbl-b antibodies. Finally, cells expressing each construct (typically 30–40% of cells) were visualized by confocal microscopy (**Figure 3C**). A quantitative assessment of the degree of colocalization between the BCR and Lamp-1 in MIGR1 infected *Cblb^−/−^* cells is provided in **Figure 3D** (n = 3 experiments). As can be seen, retroviral mediated expression of WT Cbl-b reconstituted BCR trafficking to late endosomes, while there was no reconstitution mediated by vector alone. Examination of the various mutants indicated that the C-terminal domain, which lacks the tyrosine kinase-binding (TKB) domain, could reconstitute BCR trafficking as could a Cbl-b molecule lacking E3 activity (Cbl-b^C373A^). In contrast, a mutant lacking the C-terminal UBA domain could not. Interestingly, expression of Cbl-b^GY**Δ**AQ^, in which the Ub binding motif within the UBA domain [20], [29] was mutated, reconstituted BCR trafficking. These data indicate that the Cbl-b UBA domain, but not the ability of the UBA domain to bind ubiquitin, is necessary for BCR endocytic trafficking.

**Figure 4:**
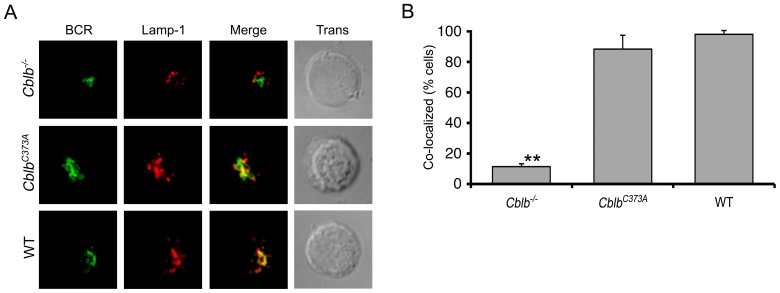
BCR endocytic trafficking is normal in splenocytes expressing an E3 ligase dead mutant of Cbl-b (*Cblb^C373A^*). Indicated splenocytes were stimulated through the BCR and imaged after 30(A) Representative images. (B) Quantitative analysis of three independent experiments (p<0.001 for *Cblb^−/−^* vs *Cblb^C373A^* or WT).

To confirm that the E3 activity of Cbl-b was dispensable for BCR endocytic trafficking, we obtained mice in which gene targeting had been used to derive mice expressing Cbl-b bearing the C373A mutation [18]. Spleens from these mice or WT controls were harvested and splenic B cells isolated and stimulated as described above. Aliquots were then fixed, stained, and visualized by confocal microscopy. Representative images are provided in **Figure 4A**, and quantitative assessments from three independent experiments are depicted in **Figure 4B**. These data indicate that BCR endocytic trafficking was similar in WT and *Cblb^C373A^* B splenocytes. These results indicate that, although Cbl-b contributes to the ubiquitination of Igβ, this function is dispensible or redundant for BCR endocytic trafficking.

Our data indicated that the carboxyterminal domain of Cbl-b, which contains the UBA domain, was required for BCR endocytic trafficking. This finding was potentially revealing as the amino acid sequences of c-Cbl and Cbl-b are divergent in the UBA domain. Therefore, we next examined if ectopic expression of c-Cbl in *Cblb^−/−^* splenocytes could restore BCR endocytic trafficking.

**Figure 5:**
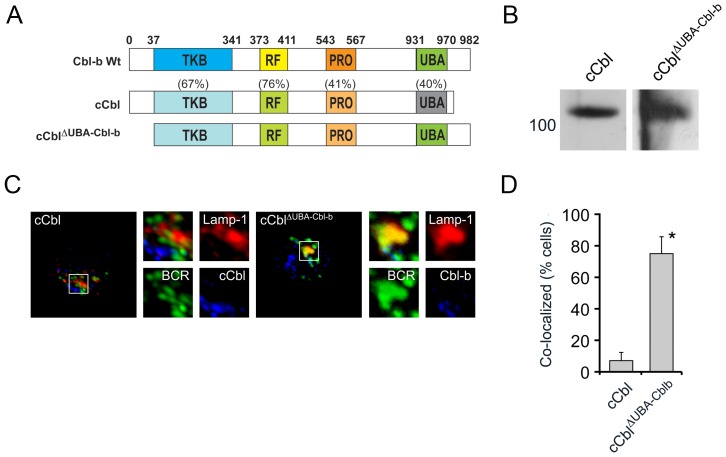
The UBA domain of Cbl-b, in the context of c-Cbl, is sufficient for BCR endocytic trafficking. (A) Schematic representation of Cbl-b and c-Cbl encoding constructs used to reconstitute *Cblb^−/−^* splenocytes. Percentages represent homology between c-Cbl and Cbl-b in indicated domains. (B) Immunoblot of PlatE cell lysates expressing indicated constructs with anti-c-Cbl antibodies. (C) Representative confocal images of *Cblb^−/−^* splenocytes reconstituted with virus encoding c-Cbl or c-Cbl**^Δ^** ^UBA-Cbl-b^ after stimulation through BCR for 30 minutes (n = 3). (D) Quantitation of experiments provided in (C, BCR co-localization with Lamp-1) across three independent experiments (30 cells/exp) (p<0.001).

A cDNA encoding WT c-Cbl (**Figure 5A**) was cloned into MIGRI and packaged in PlatE cells (anti-c-Cbl immunoblots provided in **Figure 5B**). Packaged virus was used to infect *Cblb^−/−^* splenocytes which were assayed by confocal microscopy (**Figure 5C**) and scored for reconstitution of BCR endocytic trafficking (**Figure 5D**) as described above. As can be seen, ectopic expression of c-Cbl did not reconstitute BCR endocytic trafficking in *Cblb^−/−^* B splenocytes. In keeping with these results, confocal microscopic analysis of WT splenocytes revealed that, in contrast to Cbl-b, c-Cbl was not recruited to the ligated BCR complex (**Figure S2**).

We next examined if the UBA domain of Cbl-b, in the context of c-Cbl, was sufficient to reconstitute BCR endocytic trafficking. To this end, we constructed a cDNA encoding a chimeric c-Cbl molecule in which the UBA domain of c-Cbl was replaced with that of Cbl-b (c-Cbl**^Δ^** ^UBA-Cbl-b^)(**Figure 5A–D**). This chimeric molecule was then expressed in *Cblb^−/−^* splenocytes and cells were analyzed by confocal microscopy. Expression of c-Cbl**^Δ^** ^UBA-Cbl-b^ restored normal BCR endocytic trafficking. These observations indicate that the unique ability of Cbl-b to mediate endocytic trafficking is fully encoded within the carboxyterminal region containing the UBA domain.

### Cbl-b is required for TLR9 endocytic trafficking and for TLR9-dependent B cell responses in vivo

**Figure 6:**
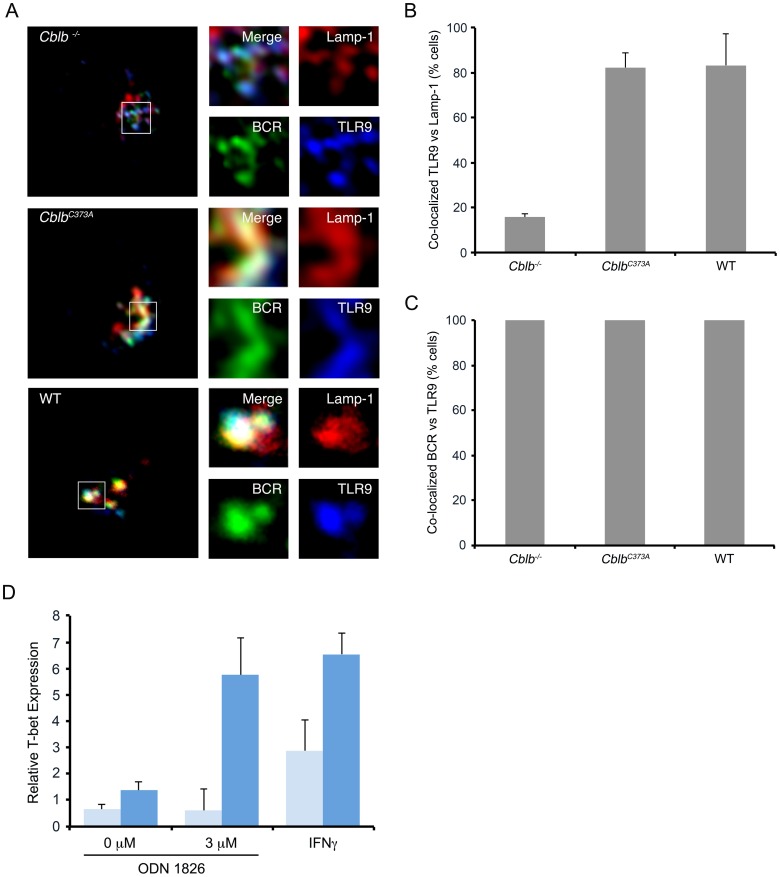
Cbl-b is required for the endocytic transit of TLR9. (A) Representative confocal microscopic images of splenocytes from mice with indicated genotypes. For experiments, cells were stimulated through the BCR (green) for 30 minutes then fixed and stained for TLR9 (blue) and Lamp-1 (red)(n = 3). (B) Quantitation of experiments shown in (A) for fraction of cells demonstrating significant co-localization of TLR9 with Lamp-1 (n = 3, 30 cells/exp) (Cbl-b^−/−^ vs. Cblb^C373A^ or WT, p<0.001). (C) Quantitation of fractions of cells in (A) demonstrating significant co-localization between BCR and TLR9 (n = 3, 30 cells/exp). (D) *In vitro* assay of T-bet induction in response to ODN 1826 or control ODN targeted through the BCR (n = 3, p<0.01).

In addition to inducing rapid endocytic transit of the BCR, receptor stimulation induces the translocation of TLR9 into late endosomes [9], [10]. Therefore, we next examined if Cbl-b was required for BCR ligation-induced TLR9 transit. B splenocytes from WT, *Cblb^−/−^*, and *Cblb^C373A^* mice were stimulated as in Figure 1 for 30 minutes and then analyzed by confocal microscopy for the co-localization of the BCR, Lamp-1 and TLR9. As can be seen in **Figures 6A**–**6C**, in *Cblb^−/−^* mice, stimulation of the BCR induced the co-localization of the endocytosed BCR with TLR9. However, both receptors were excluded from Lamp-1^+^ late endosomes. As expected, this compartment was Cathepsin L^+^ consistent with terminal lysosomes (**Figure S3**). In contrast, both the BCR and TLR9 entered late endosomes in B splenocytes from *Cblb^C373A^* mice. No staining of TLR9 was observed in splenic B cells from *TLR9^−/−^* mice [10]. These data indicate that TLR9 has similar Cbl-b requirements as the BCR for entry into late endosomes.

As in *Cblb^−/−^* splenocytes TLR9 was sequestered within terminal lysosomes, we examined if these cells were unresponsive to TLR9 ligands delivered through the BCR. A unique downstream target of TLR9 is the transcription factor T-box expressed in T cells (T-bet), which can be directly activated by CpG via TLR9 in B cells through an IFN_γ_R/STAT1-independent pathway [30]. Therefore, WT and *Cblb^−/−^* splenic B cells were incubated with streptavidin-conjugated F(ab)_2_ anti-IgG antibodies followed by biotinylated ODN 1826 or control ODN (Invivogen). After six hours, RNA was isolated from stimulated B cells, and T-bet mRNA expression was assayed by quantitative PCR. IFN_γ_ stimulated cells were used as a positive control [30]. As can be seen in **Figure 6D**, BCR targeted ODN 1826 stimulated T-bet expression in WT cells. The control ODN did not induce T-bet expression. However, in *Cblb^−/−^* splenocytes ODN 1826 failed to induce T-bet. These data reveal a defect in T-bet responses to TLR9 ligands in *Cblb^−/−^* splenocytes. T-bet induction was also defective through IFN_γ_suggesting the presence of more global activation defects.

## Discussion

Herein, we demonstrate that recruitment of Cbl-b to the BCR is necessary for both clustering the BCR on the cell surface and for guiding internalized BCRs into late endosomes. This activity of Cbl-b was unique and could not be supplanted by c-Cbl. Furthermore, Cbl-b was required for TLR9 entry into late endosomes and for activation of TLR9 by BCR captured ligands. In the absence of Cbl-b, the BCR and TLR9 became sequestered together within terminal lysosomes. These data indicate that Cbl-b plays an important role in coupling adaptive and innate immune signaling responses in B lymphocytes.

Our findings both confirm and extend recent observations in chicken cell lines on the importance of Cbl in determining BCR endocytic fate. As observed in chicken cell lines [28], Cbl-b was required for ligand-induced clustering on the cell surface. In these cell line studies, Cbl played a role in coupling the BCR to the motor protein dynein. Dynein has also been implicated in endocytic trafficking [31] indicating that the observed defects in clustering and endocytic trafficking could reflect a common underlying mechanism.

The unique ability of Cbl-b to facilitate receptor endocytic trafficking mapped to the Cbl-b carboxyl-terminal tail that contains the UBA domain. One well-described difference between Cbl-b and c-Cbl is that the Cbl-b UBA domain can bind Ub while the c-Cbl UBA cannot. Ubiquitin binding mediates Cbl-b dimerization and increases E3 ligase activity [29]. However, the ability of Cbl-b to enable BCR endocytic trafficking to late endosomes was not dependent on the Cbl-b ubiquitin-binding motif. In chicken B cells, c-Cbl is recruited to the BCR and is required for BCR surface clustering [28]. Chicken c-Cbl is also predicted to not bind Ub, and therefore, there is likely another unidentified functional domain, conserved between chicken c-Cbl and human and murine Cbl-b, that enables BCR endocytic trafficking.

Previous publications have focused on Cbl-b as a negative regulator of lymphocytes [32]. Mice deficient in Cbl-b develop an autoimmune syndrome associated with lymphocytic infiltrates in multiple organs [33], and they are highly susceptible to experimental autoimmune encephalomyelitis [34] and collagen induced arthritis [35]. *Cblb^−/−^* T cells do not need CD28 costimulation for activation and cannot be tolerized [34]. These phenotypic changes have been related to Cbl-b-mediated regulation of PI3K [36] and PLC_γ_1 [35], respectively. In B cells, Cbl-b negatively regulates signaling through CD40 [37] and has been demonstrated to bind and ubiquitinylate multiple proximal BCR signaling components [33], [38]–[41]. Our observation that Cbl-b is recruited to the aggregated BCR complex provides a mechanism by which Cbl-b could gain access to many of these signaling substrates.

However, given the multiple negative signaling functions ascribed to Cbl-b, the phenotype of *Cblb^−/−^* mice is relatively mild. Autoimmunity does not develop until after six months of age, and potentially pathogenic immune complex deposits in glomeruli are only observed in some aged mice (10 months old) in which both *Cbl* and *Cblb* had been targeted in B cells [42]. By ELISA, anti-dsDNA antibodies are detected in *Cblb^−/−^* mice. However, such ELISAs can be falsely positive and are not as relevant as *Crithidia luciliae* immunofluorescence (IF) assays. In fact, in *MRL/Mp^lpr/lpr^* mice, TLR9 deficiency greatly diminishes anti-dsDNA responses as measured by IF but not as measured by ELISA [43]. Thus, the available evidence indicates that the intrinsic B cell defect in *Cblb^−/−^* mice is not severe. We propose that this is because Cbl-b is a complex molecule with both negative and positive effects on peripheral B cell activation.

While the Cbl-b UBA domain is uniquely required for BCR endocytic trafficking, it is likely that one or more additional Cbl-b domains, conserved in c-Cbl, contribute by linking the receptor to downstream effector mechanisms. When tyrosine phosphorylated, Cbl proteins bind to CD2AP (CD2 adaptor protein) and the homolog CIN85 [44]–[46]. CIN85 has mostly been studied in the context of epidermal growth factor receptor (EGFR) trafficking, while CD2AP appears to play a prominent role in TCR function [47]. CIN85 can localize to late endosomes [48], and has been implicated in the endocytic degradation of the EGFR [46]. T cells from *CD2AP^−/−^* mice have a defect in ligand-induced TCR degradation that appears to be due to a block in trafficking to lysosomes [47]. The exact function of CD2AP and CIN85 is not known, although the latter has been demonstrated to function as a scaffold for multiple molecules implicated in endocytic trafficking [49].

Cbl-b and c-Cbl likely share another function important for BCR endocytic trafficking. The region immediately carboxyterminal to the RF domain (the RF tail) mediates phosphorylation and degradation of Hrs [50]. Mutation of this domain both abrogates Hrs phosphorylation and prevents fusion of EGFR-containing early endosomes with acidic late endosomes. This phenotype is similar to that observed for BCR trafficking in *Cblb^−/−^* splenocytes.

Surprisingly, Cbl-b was also required for entry of TLR9 into late endosomes. It is possible that Cbl-b regulates one or more signaling pathways mediating TLR9 transit into late endosomes. This possibility is consistent with our findings in anergic B cells that defective JNK activation prevents the transit of both the BCR and TLR9 into late endosomes [10]. It is also consistent with the apparent dependency of MIIC maturation on Cbl-b and with the general known interplay between BCR endocytic trafficking and intracellular signaling [9]. Alternatively, Cbl-b could provide a necessary linker or scaffolding function for TLR9. As Cbl-b is recruited to the BCR, such a model would require that the BCR and TLR9 come into close proximity before entry into late endosomes. The strong co-localization observed between the BCR and TLR9 in BCR-stimulated *Cblb^−/−^* splenocytes indicates that this is the case.

By demonstrating a unique, non-E3 dependent function for Cbl-b, our data provide insights into how the family of Cbl ligases may mediate different cellular functions. Deletion of Cbl-b results in immune hyperactivity and some features of autoimmunity. In this regard, Cbl-b is broadly similar to c-Cbl. However, specific mechanistic dissection indicates that the *Cblb^−/−^* phenotype reflects the net effect of deleting a molecule that can both enable and suppress specific immune mechanisms. Our data also demonstrate that Cbl-b is more than a biochemical component of several signaling pathways. Rather, it performs an important cell biological function by coordinating the intracellular movement of immune recognition receptors. Therefore, Cbl-b functions in two or more fundamental cellular processes that determine immune responses to both self and non-self antigens.

## Supporting Information

Figure S1 **Normal trafficking of the ligated BCR to early endosomes in** ***Cblb^−/−^*** **splenocytes.** (A) *Cblb^−/−^* or WT splenocytes were stimulated as in Figure 1 for 15 or 45 minutes and then fixed and stained with antibodies to the early endosomal marker EEA1 (C45B10, Cell Signaling). Shown are representative images of results obtained from three independent experiments. In (B) is a quantitation of the co-localization between the BCR and EEA1 in *Cblb^−/−^* (light grey) and WT (dark grey) (n = 3 experiments, *p = 0.002).
(TIF)

Figure S2 **C-Cbl is not recruited to the aggregated BCR complex.** Cells were stimulated for 0 to 20 minutes through the BCR, fixed, stained with either anti-Cbl-b or anti-c-Cbl antibodies and analyzed as in Figure 2. In (A) is provided representative images obtained two minutes after stimulation (n = 3). In (B) is a quantitation of the co-localization between the BCR and either c-Cbl (red, squares) or Cbl-b (blue, diamonds)(n = 3).
(TIF)

Figure S3 **Both the BCR and TLR9 target the same Lamp-1^−^Cathepsin L^+^ compartment.** (A) Representative four color confocal micrographs of WT and *Cblb^−/−^* splenocytes stimulated with TexasRed-conjugated anti-BCR antibodies for 30 minutes. Cells were then fixed, stained with antibodies specific for TLR9, Cathepsin L and Lamp-1 and visualized by confocal microscopy. (B) Quantification of co-localization between different markers in WT (dark grey) and *Cblb^−/−^* (light grey) splenocytes. (n = 3, *p = 8.65×10^−6^, **p = 0.0015 and ***p = 0.0005).
(TIF)
