# Supplementary figures and images for: Recruitment of Cbl-b to B Cell Antigen Receptor Couples Antigen Recognition to Toll-Like Receptor 9 Activation in Late Endosomes
Source: PLoS One. 2014 Mar 20;9(3):e89792. doi: 10.1371/journal.pone.0089792 (PMC3961229; doi:10.1371/journal.pone.0089792)

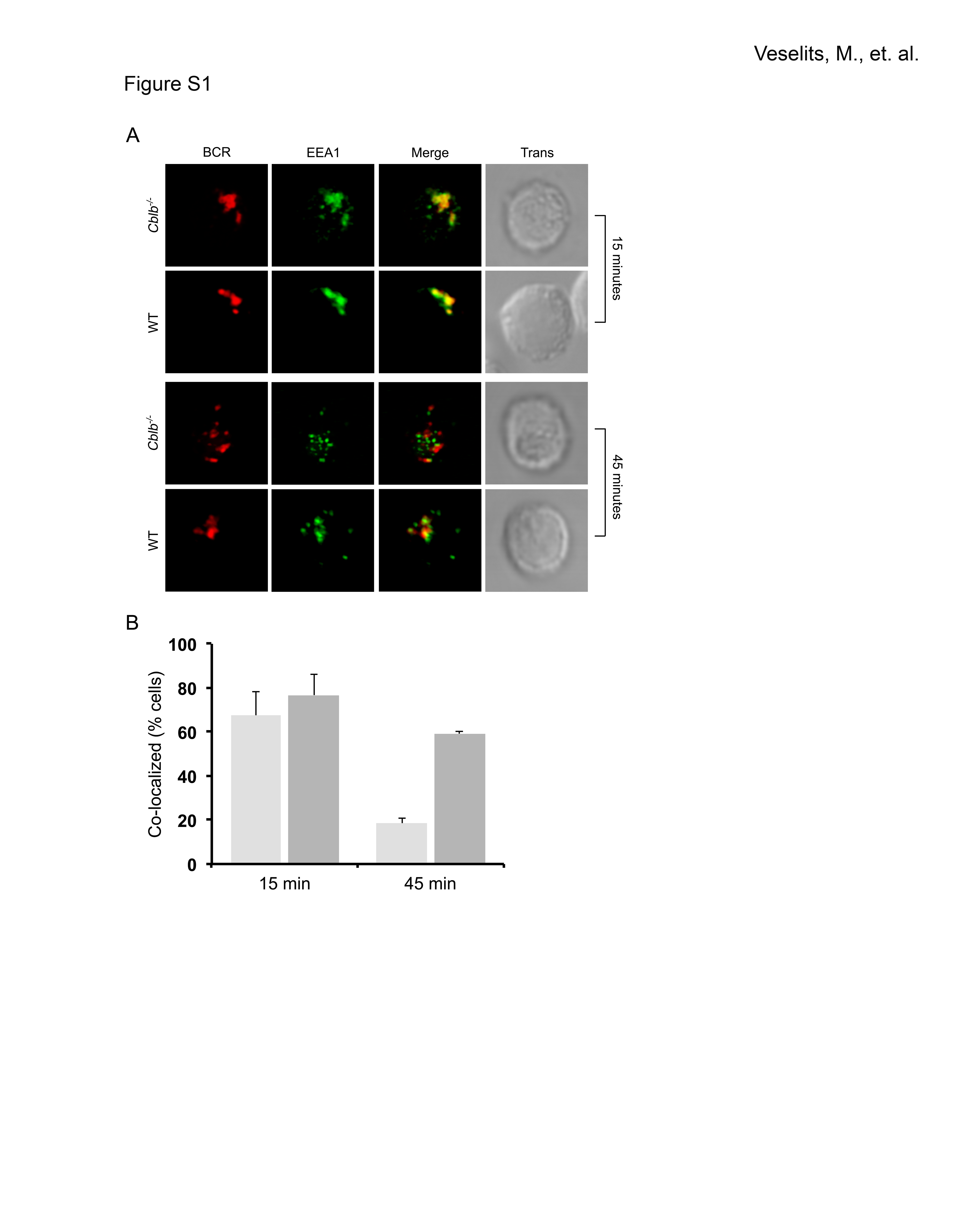

Supplement: Figure S1 — Normal trafficking of the ligated BCR to early endosomes in Cblb−/− splenocytes. (A) Cblb−/− or WT splenocytes were stimulated as in Figure 1 for 15 or 45 minutes and then fixed and stained with antibodies to the early endosomal marker EEA1 (C45B10, Cell Signaling). Shown are representative images of results obtained from three independent experiments. In (B) is a quantitation of the co-localization between the BCR and EEA1 in Cblb−/− (light grey) and WT (dark grey) (n = 3 experiments, *p = 0.002). (TIF) [file pone.0089792.s001.tif]

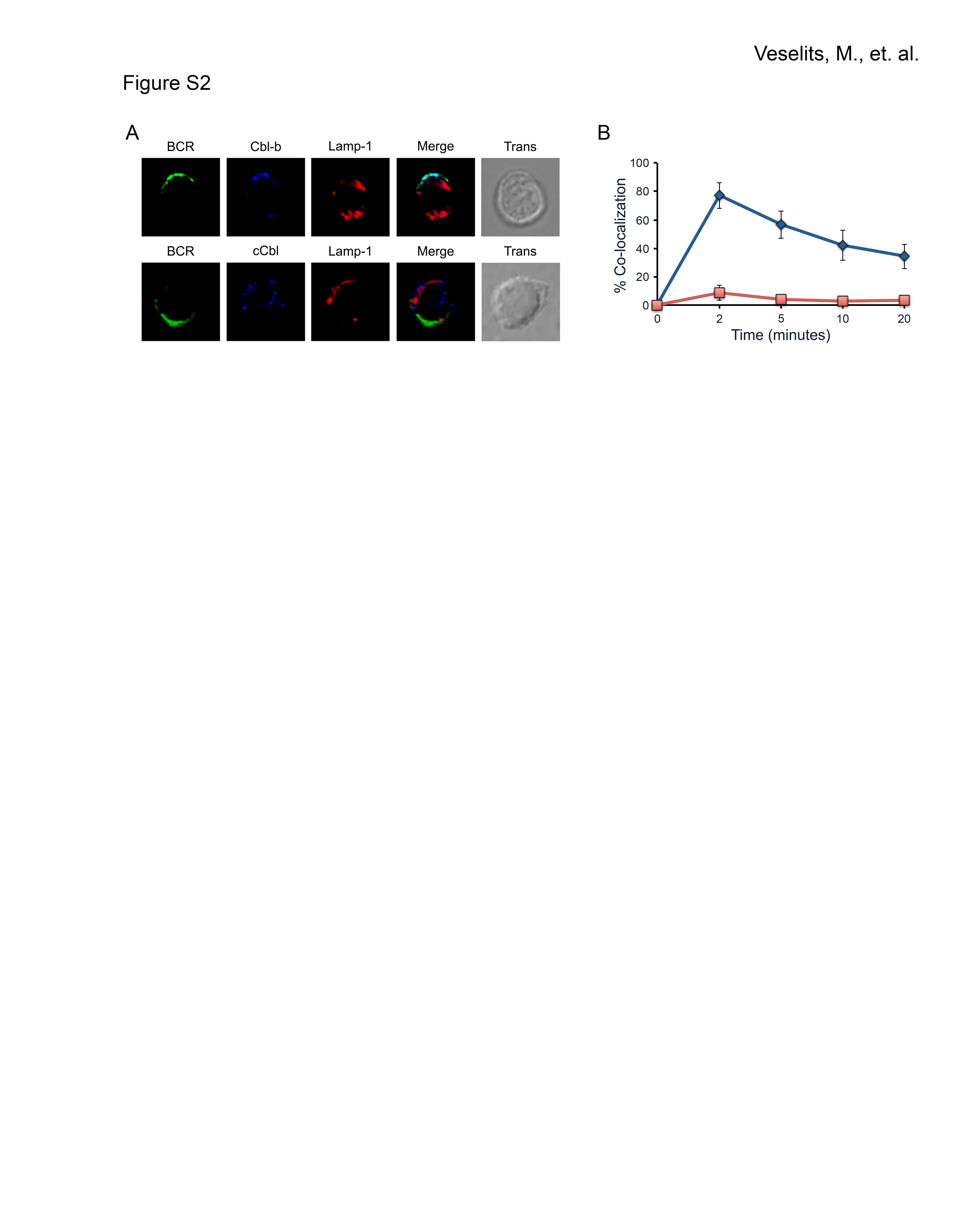

Supplement: Figure S2 — C-Cbl is not recruited to the aggregated BCR complex. Cells were stimulated for 0 to 20 minutes through the BCR, fixed, stained with either anti-Cbl-b or anti-c-Cbl antibodies and analyzed as in Figure 2. In (A) is provided representative images obtained two minutes after stimulation (n = 3). In (B) is a quantitation of the co-localization between the BCR and either c-Cbl (red, squares) or Cbl-b (blue, diamonds)(n = 3). (TIF) [file pone.0089792.s002.tif]

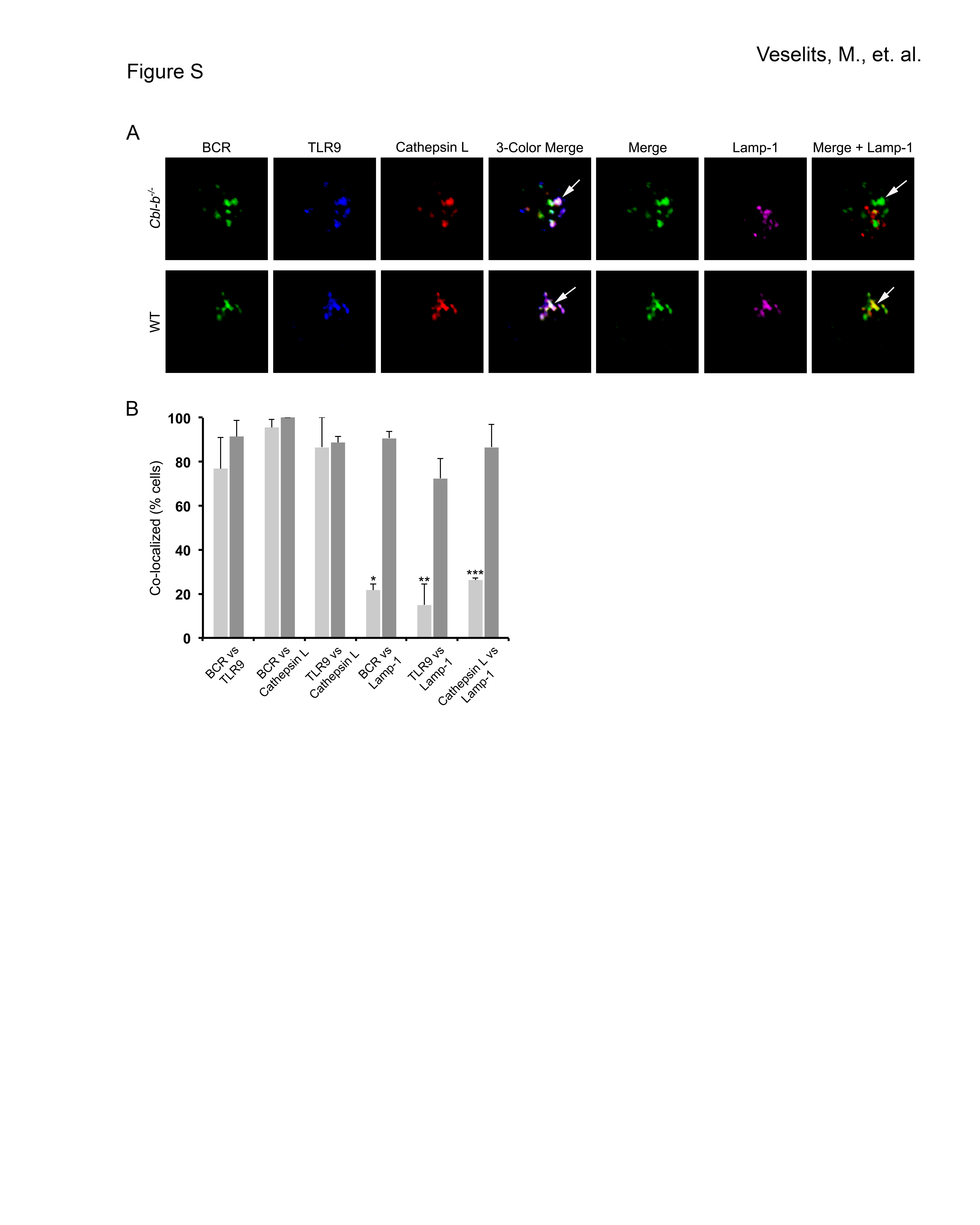

Supplement: Figure S3 — Both the BCR and TLR9 target the same Lamp-1−Cathepsin L+ compartment. (A) Representative four color confocal micrographs of WT and Cblb−/− splenocytes stimulated with TexasRed-conjugated anti-BCR antibodies for 30 minutes. Cells were then fixed, stained with antibodies specific for TLR9, Cathepsin L and Lamp-1 and visualized by confocal microscopy. (B) Quantification of co-localization between different markers in WT (dark grey) and Cblb−/− (light grey) splenocytes. (n = 3, *p = 8.65×10^−6^, **p = 0.0015 and ***p = 0.0005). (TIF) [file pone.0089792.s003.tif]
